# Dual-Mode Solution Plasma Processing for the Production of Chitosan/Ag Composites with the Antibacterial Effect

**DOI:** 10.3390/ma13214821

**Published:** 2020-10-28

**Authors:** Valerii Titov, Daniil Nikitin, Irina Naumova, Nikolay Losev, Irina Lipatova, Dmitry Kosterin, Pavel Pleskunov, Roman Perekrestov, Nikolay Sirotkin, Anna Khlyustova, Alexander Agafonov, Andrei Choukourov

**Affiliations:** 1 G. A. Krestov Institute of Solution Chemistry of the Russian Academy of Sciences, Akademicheskaya 1, 153045 Ivanovo, Russia; titov25@gmail.com (V.T.); nvl@isc-ras.ru (N.L.); i_lipatova@bk.ru (I.L.); alexsad8@yandex.ru (N.S.); kav@isc-ras.ru (A.K.); ava@isc-ras.ru (A.A.); 2 Department of Macromolecular Physics, Faculty of Mathematics and Physics, Charles University, V Holešovičkách 2, 18000 Prague, Czech Republic; pleskunov@protonmail.ch (P.P.); choukourov@kmf.troja.mff.cuni.cz (A.C.); 3 Department of Natural Sciences, Ivanovo State Agricultural Academy, Sovetskaya 45, 153012 Ivanovo, Russia; irinauma@mail.ru (I.N.); d.costerin@yandex.ru (D.K.); 4 Institute of Physics of the Czech Academy of Sciences, Na Slovance 1999/2, 182 21 Prague, Czech Republic; mr.perekrestov@gmail.com

**Keywords:** chitosan, silver nanoparticles, composite, plasma-solution system, antibacterial properties

## Abstract

The development of novel biocompatible and biodegradable materials for medical applications has been drawing significant interest in the scientific community for years. Particularly, chitosan loaded with silver nanoparticles (Ag NPs) has a strong antimicrobial potential and could be applied, for example, as wound dressing material. In this work, chitosan/Ag NP composites were produced utilizing a single-step plasma-solution process, which is simple and environmentally friendly. An acetic solution of chitosan containing AgNO_3_ was treated by the direct current (DC) atmospheric pressure glow discharge, with the liquid serving as either cathode or anode. The plasma-solution system with liquid anode is more useful for the production of Ag NPs. Nevertheless, the NP size is comparable for both cases. The plasma treatment with both polarities led to chitosan degradation. The cleavage of glucosidic chains mostly occurred in the system with the liquid cathode, whereas the side oxidation reactions took place when the solution served as the anode. The oxidation processes were possibly induced by the hydrogen peroxide H_2_O_2_ efficiently formed in the last case. The composite materials produced with both polarities of liquid electrode demonstrated the bactericidal action against Gram-negative *Escherichia coli*, Gram-positive *Staphylococcus aureus*, and Gram-positive *Bacillus subtilis*.

## 1. Introduction

In recent years, there has been a noticeable advancement in the development of new materials based on natural polymers, such as starch, cellulose, chitin, or chitosan [1]. These polysaccharides are useful for various scientific and industrial applications due to their unique properties. For example, the superior adsorption properties make polysaccharides highly attractive for water purification from organic and inorganic pollutants, such as dyes and heavy metals [2,3,4,5,6]. Moreover, cellulose can be utilized to produce “smart materials”, including active electronic papers, sensors, shape-memory materials, and smart membranes [7]. Chitin and chitosan (product of the deacetylation of chitin), which are non-toxic, biocompatible, and biodegradable, have been successfully applied in food technology [8] and agriculture [9]. The biological activity of chitosan strongly depends on the molecular weight and the degree of deacetylation. For instance, low molecular weight chitosan (50–150 kDa) has demonstrated bactericidal action against several dangerous pathogens, including *Staphylococcus aureus* and *Escherichia coli*, unlike the higher molecular weight chitosan (>150 kDa) [10]. At the same time, chitosan with a higher degree of deacetylation (>95%) is preferable for cell growth [11]. Thereby, the biomedical applications of chitosan require careful adjustment of both parameters. Chitosan has already been successfully utilized in dental applications [12], bone tissue engineering [13], drug delivery systems [14], and wound healing [15]. However, bacterial evolution and biofilm formation force scientists to develop novel materials with improved bactericidal activity. In particular, the antimicrobial efficiency of chitosan-based materials (foils, fibers, membranes, or scaffolds) can be enhanced by their loading with a potent bactericidal agent, such as silver nanoparticles (Ag NPs).

The disinfection effect of silver has been well known for a long time. Currently, silver coatings are utilized in medicine primarily for the fabrication of wound dressing materials. Most research studies describe the antibacterial effect of silver in terms of the mechanism of Ag^+^ ion release [16]. The ion release can be substantially enhanced if silver is used in a nano-dispersed form due to its large surface area. For example, clinical studies have demonstrated no wound pus drainage for post-surgery patients treated with Ag NPs-containing gel [17]. Moreover, the traditional bandages made of silk, cotton, or cellulose can be impregnated with NPs to significantly improve their action [18,19]. Nevertheless, the use of free Ag NPs should be carefully pondered because the NPs may exhibit high toxicity toward the human cells. To prevent the direct contact of NPs with human cells, they are incorporated or covered by a biocompatible polymer matrix. Among different polymers, chitosan seems to be one of the most suitable candidates, also because of the proven synergistic antibacterial action in combination with Ag ions [20,21].

Composites of chitosan with metal NPs were reported for the first time at the beginning of the 2000 s [22]. The NPs of silver, gold, platinum, and palladium were obtained by chemical reduction of metal salts added to the chitosan solution. The composites were produced by casting and drying of the resulting mixture on glass substrates. It is worth noting that Ag NPs were usually produced by the chemical reduction of silver nitrate AgNO_3_, the concentration of which regulated the number of NPs in the composite. The wet chemical method became the most frequently used to fabricate chitosan-based hybrid materials due to its simplicity [23,24,25,26,27,28]. A comparative study revealed that the bactericidal and antifungal action of Ag NPs-loaded chitosan is higher than chitosan itself [29]. Moreover, the antibacterial effect can be enhanced by introducing an additional bactericidal agent, for example, zinc oxide (ZnO) [30,31,32]. Foils are the simplest materials that can be obtained from the chitosan/Ag NPs solution. The development of electrospinning technology provided an opportunity to obtain more complex forms of composite, such as fibers and membranes [33,34]. The safety of such composites from the cytotoxicity point of view has been confirmed by several studies [35,36,37]. Despite all benefits, the wet chemical method has serious drawbacks, including the long NPs formation (typically several hours) and high reagent consumption. Therefore, new green methods to produce chitosan/Ag NPs composites have become one of the topical issues.

Plasma-chemical processes are known as a good alternative. In particular, the reduction of AgNO_3_ with the formation of Ag NPs can be induced by the plasma action. Non-equilibrium plasma produces different types of active species, such as solvated electrons, radicals, ions, etc., which can catalyze the cations’ reduction and accelerate NPs growth. Different types of discharges have been applied for the production of Ag NPs, including dielectric barrier discharge [38], plasma jet [39], pulsed microwave discharges [40,41], or microdischarge with liquid anode [42]. In all these systems, the NPs formation time was reduced to minutes instead of hours in equilibrium wet chemistry.

Plasma processing of chitosan has also been under the scrutiny of scientists for years. In particular, the plasma treatment of chitosan solution has been successfully applied for the tunable decomposition of chitosan, accompanied by the cleavage of glycosidic chains and the formation of lower molecular weight species [43,44,45]. These processes are mediated by the formation of radicals and can be applied for the fast and effective immobilization of chitosan onto polymeric supports for potential wound dressing applications [46,47].

Despite extensive research on either chitosan or Ag NP solution plasma processing, only a few papers have been published on the combined plasma-based production of chitosan/Ag NP composites. For example, Ar plasma post-treatment of electrospun chitosan fibers loaded with AgNO_3_ led to the reduction of the latter with the formation of nm-sized Ag NPs [48]. Plasmas in liquid or in contact with liquid have also been investigated; the discharge between metal electrodes submerged in chitosan/AgNO_3_ solution or the gas discharge sustained between the outer electrode and liquid surface has been utilized for facile synthesis of chitosan/Ag NPs composites [49,50].

In this paper, we use a system similar to one presented in [46], in which the direct current (DC) plasma is generated between the point electrode and the surface of the liquid. Unlike previous studies, we lay emphasis on how the electrode polarity influences the chemical properties, the structure, and the antimicrobial action of the resultant chitosan/Ag NP composites.

## 2. Materials and Methods

### 2.1. Materials

The solutions of chitosan and silver nitrate (AgNO_3_) were prepared separately. Chitosan (Bioprogress Ltd., Shchyolkovo, Russia), molecular weight 192 kDa, degree of deacetylation 82%) with a mass of 1 g was dissolved in 49 g of 4% (*w*/*v*) of acetic acid solution (Chimmed Ltd., Moscow, Russia, GOST 61-75). AgNO_3_ powder (Sigma-Aldrich, Moscow, Russia) with a mass of 0.13 g was dissolved in 50 g of deionized water. The AgNO_3_ solution was added dropwise to the acetic solution of chitosan at constant stirring to obtain a working solution in which the chitosan concentration was 1% (*w*/*v*). The working solution was maintained at room temperature of 25 °C and constant stirring for 5 h, according to [51].

### 2.2. Experimental Setup

**Figure 1:**
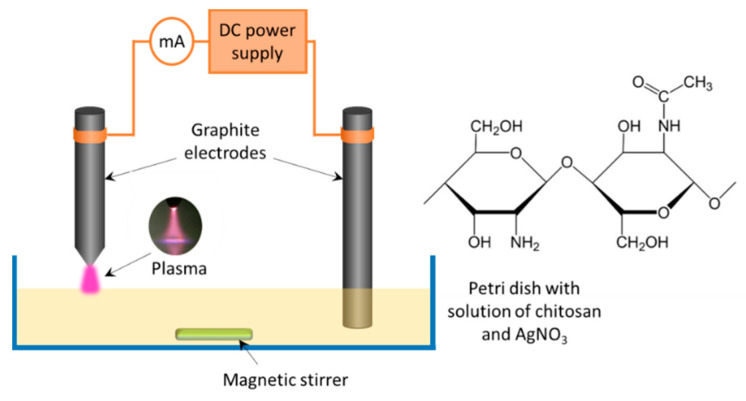
Scheme of the experimental setup and chitosan chemical structure.

The experimental setup used for the solution plasma treatment is presented in Figure 1.

A Petri dish was filled with 50 mL of the working solution. A graphite electrode was immersed in the solution, and another pointed graphite electrode was positioned in the gas phase at 2 mm above the solution surface. The polarity of the electrodes could be changed. Atmospheric pressure discharge was ignited using a self-constructed DC power supply at 45 mA current. The treatment time was varied from 2 to 30 min. The maximal temperature of the solution during plasma processing reached 81 °C.

An iodometric titration established the concentration of H_2_O_2_ in the chitosan/AgNO_3_ solution after the plasma treatment with the addition of ammonium molybdate, which served as a selective catalyst for the reaction of H_2_O_2_ with the iodide ions [52].

The plasma-treated solutions were filtered through the filter paper and cast on round glass substrates (diameter 80 mm). The samples were dried for 48 h under ambient conditions (25 °C, relative humidity 40%) to evaporate the solvent. The resulting chitosan/Ag composite foils were mechanically separated from the substrates and rinsed by the deionized water to remove the residues of acetic acid and AgNO_3_.

### 2.3. Analysis of Chitosan/Ag NP Solutions

The average molecular weight (M) of chitosan was evaluated from the intrinsic viscosity of the chitosan solution using a Kuhn–Mark–Houwink equation, [η] = *KM*^α^. Here, [η] is the intrinsic viscosity and K and α are constants. The intrinsic viscosity was measured experimentally by a capillary-based method using an Ubbelohde viscometer (Cannon Instrument Company, State College, PA, USA) with a capillary 0.56 mm in diameter. The chitosan solutions with different concentrations were prepared by a sequential dilution of the initial solution with 2% acetic acid in a buffer of sodium acetate (0.2 M, pH = 5.4). The specific viscosity of these solutions was measured at 25 °C, and the intrinsic viscosity was determined by performing the extrapolation. The values of *K* = 1.464 × 10^-4^ and α = 0.885 for chitosan in acetate buffer solutions were taken from [53].

UV–VIS spectra of solutions were acquired using a SF-56 Lomo spectrophotometer (Saint Petersburg, Russia) in the range of 190–700 nm. Quartz cuvettes with an optical path length of 10 mm were used. The 2% (*w*/*v*) solution of acetic acid was used as a reference. Chitosan solutions were prediluted by a factor of 5 and 50 with 2% acetic acid before the measurements.

### 2.4. Characterization of Chitosan/Ag NP Composite Foils

The morphology of chitosan/Ag NP composite foils was studied using a scanning electron microscope (SEM, Tescan Mira III, Brno, Czech Republic) equipped with an energy-dispersive X-ray spectroscopy (EDX) module for the chemical analysis. The SEM measurements were performed with an accelerating voltage of 10 kV in secondary electron and backscattered electron modes.

Additionally, the chemical composition of chitosan/Ag foils was investigated using X-ray photoelectron spectroscopy (XPS). An Al Kα X-ray source (1486.6 eV, Specs, Berlin, Germany) with a multi-channel hemispherical electrostatic analyzer (Phoibos 100, Specs, Berlin, Germany) was used. Survey and high-resolution spectra were acquired with a pass energy of 40 and 10 eV, respectively. The spectra were charge-corrected using the position of aliphatic carbon at 285.0 eV. Fourier-transform infrared spectroscopy was carried out in a single attenuated total reflectance mode (FTIR-ATR) using a NICOLET 6700 FTIR, (Thermo Fisher Scientific, Waltham, MA, United States) spectrometer with a ZnSe crystal. Sixteen scans with a spectral resolution of 4 cm^−1^ were co-added to achieve a good signal-to-noise ratio. All the measurements were performed at room temperature.

The information about the crystalline structure of the samples was obtained from X-ray diffraction (XRD). The XRD measurements were performed using a Philips X’Pert PRO MRD diffractometer (Cu Kα radiation, λ = 0.15418 nm, PANalytical B.V., Almelo, Netherlands) in a Bragg–Brentano geometry.

Ag NPs formed during the solution plasma treatment were characterized by transmission electron microscopy (TEM JEOL 1200 EX, Tokyo, Japan). Carbon-coated copper grids (Agar Scientific, Stansted, Essex, UK) were immersed in the plasma-treated solution for 30 min. Afterward, the grids were extracted, rinsed by the deionized water, and dried for 24 h under vacuum conditions.

### 2.5. Evaluation of the Antibacterial Activity of Chitosan/Ag NP Composites

The bactericidal activity of the chitosan/Ag NP composite foils was evaluated by a disc diffusion agar test against the following three types of pathogens: Gram-negative *Escherichia coli*, Gram-positive *Staphylococcus aureus*, and Gram-positive *Bacillus subtilis*. The composite samples were placed into the Petri dish with agar (Sigma-Aldrich, Moscow, Russia) contaminated with bacteria solution (concentration 10^9^ CFU/mL). The incubation was performed in a thermostat for 24 h at 37 °C. The bactericidal activity of the samples was evaluated following a Kirby–Bauer disk diffusion susceptibility test protocol [54] by measuring the diameter of the zone of inhibition.

## 3. Results

### 3.1. Plasma Treatment of the Chitosan/AgNO_3_ Solution

**Figure 2:**
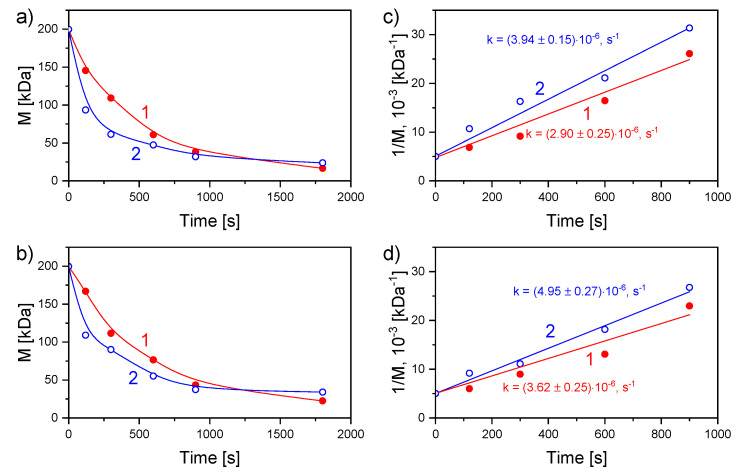
Dependencies of the viscosity average molecular mass and inverse viscosity average molecular mass of chitosan on the time of treatment by discharge with (**a**,**c**) liquid anode and (**b**,**d**) liquid cathode. 1, blank chitosan solution; 2, chitosan/AgNO_3_ solution.

The plasma treatment results in a continuous decrease in the viscosity of the solution under both polarities used. The effect is associated with the destruction of the chitosan macrochains via the cleavage of *β*-1-4-glycosidic bonds in reactions with OH radicals, also accompanied by the opening of pyranose rings [42]. Figure 2a,b shows more than a 5-fold decrease of the average molecular mass of chitosan after 1800 s of the solution plasma processing with both polarities.

A similar effect was observed in our previous work in which only one polarity with liquid cathode was used [45]. The drop in *M* occurs faster in the presence of AgNO_3_. This effect is manifested via the degradation rate constants calculated by the linear approximation according to the following equation (Figure 2c,d), Equation (1):

*1/M* = *1/M*_0_ + *kt/m* (1)

where *M*_0_ is the initial viscosity average molecular mass of chitosan, *k* is the destruction rate constant, *m* is the molecular mass of the monomer unit, and *t* is the treatment time. For both polarities, the degradation rate constants of chitosan are higher with Ag^+^ than without. In the case of the liquid anode, the values with and without AgNO_3_ are 3.94 ± 0.15 × 10^−6^ s^−1^ and 2.90 ± 0.25 × 10^−6^ s^−1^, respectively. For the discharge with the liquid cathode, the values with and without AgNO_3_ are 4.95 ± 0.27 × 10^−6^ s^−1^ and 3.62 ± 0.25 × 10^−6^ s^−1^, respectively.

**Figure 3:**
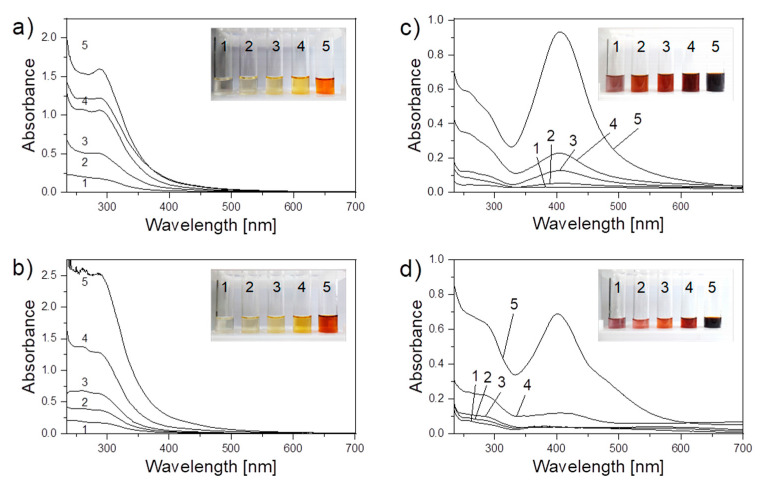
UV–VIS spectra of blank chitosan solution (left) and chitosan/AgNO_3_ solution (right) after the plasma processing by discharge with (**a**,**c**) liquid anode and (**b**,**d**) liquid cathode during the following: 1, 0 min; 2, 5 min; 3, 10 min; 4, 15 min; and 5, 30 min. The photos of solutions as they are attached as inserts.

The degradation of chitosan was accompanied by the darkening of the solution from pale yellow to saturated brown, as shown in inserts to Figure 3.

UV–VIS spectrophotometry was applied to characterize the chemical transformations that occur with chitosan in solution under the plasma action. Figure 3a,b demonstrates the evolution of the UV–VIS spectrum of the blank chitosan solution during the plasma processing in the system with the liquid anode and cathode, respectively. The plasma-solution treatment results in the intensification of the absorption in the UV region corresponding to the overlapped bands with maxima at 265 nm and 295 nm. This effect can be explained by the degradation processes taking place during the plasma treatment. The signal at 295 nm is associated with carboxyl groups at the ends of chitosan chains that appear due to the cleavage of glycosidic linkages. Another band with a maximum at 265 nm is related to carbonyl groups formed as a result of side oxidation reactions. 

Figure 3c,d shows the UV–VIS spectra of chitosan solutions containing AgNO_3_ treated by plasma using both polarities of the liquid electrode. After the plasma processing, a peak with a maximum at 410 nm appeared for both polarities. This peak is assigned to the localized surface plasmon resonance (LSPR) of Ag NPs, the effect which characterizes the collective oscillation of electrons in metals [55]. The LSPR peak in the spectra indicates the formation of Ag NPs in chitosan/AgNO_3_ solutions under the plasma action. It is worth noting that, in the system with the liquid anode, the LSPR signal appears after 5 min of plasma processing, whereas in the system with the liquid cathode, it appears only after 15 min. After 30 min of plasma treatment, the resulting intensity of the LSPR peak is higher for the liquid anode.

**Figure 4:**
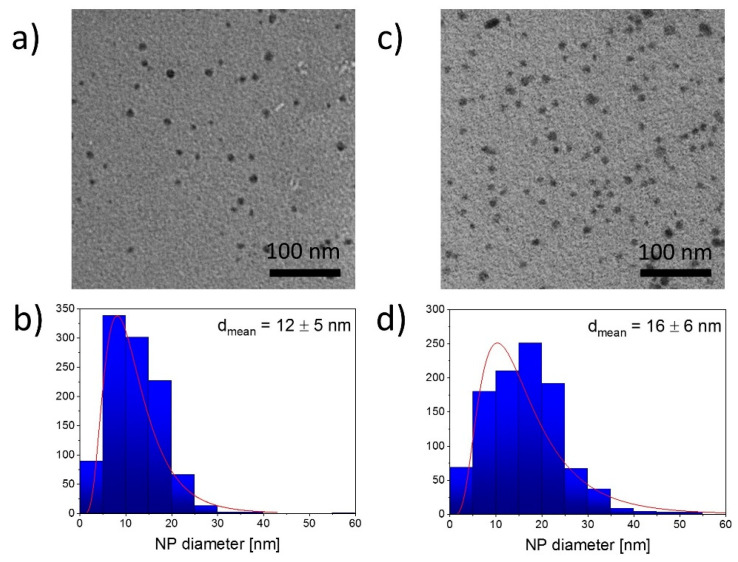
(**a**,**c**) TEM images and (**b**,**d**) size distributions of silver nanoparticles (Ag NPs) prepared using the plasma-solution processes with the liquid cathode (left) and liquid anode (right). Treatment time, 30 min.

The morphology and the size of Ag NPs were determined by TEM. The TEM images of NPs, as well as their size distributions, are shown in Figure 4.

It can be seen that Ag NPs produced with both polarities of the liquid electrode are mostly spherical. The process performed with the liquid cathode resulted in NPs with a mean diameter of 12 ± 5 nm. In the system with the liquid anode, the mean diameter of NPs was 16 ± 6 nm. It is worth noting that the size distribution of the Ag NPs prepared in this system is wider.

### 3.2. Structure and Chemical Composition of Chitosan/Ag NP Composites

**Figure 5:**
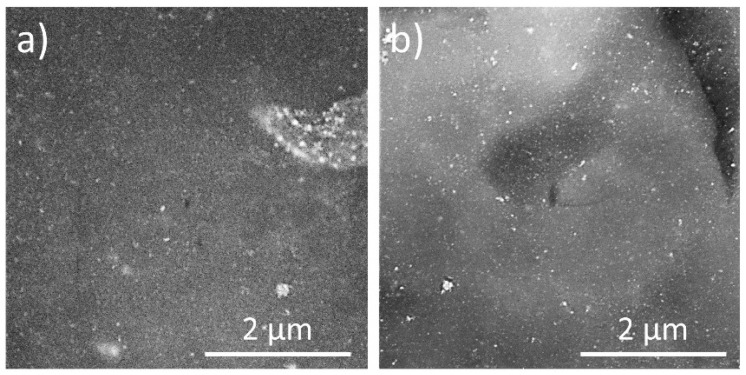
SEM images of the surfaces of chitosan/Ag NP composites prepared using a plasma-solution system with (**a**) liquid cathode and (**b**) liquid anode. Images were obtained in backscattered electron mode. Treatment time, 30 min.

Chitosan/Ag NPs composites were produced by casting the plasma-treated solutions on glass substrates with subsequent drying. The morphology of final foils was studied using SEM measurements performed in the backscattered electron mode. As shown in Figure 5, blends prepared both with liquid cathode and anode have smooth surfaces with randomly distributed Ag NPs (bright dots).

The agglomerates of Ag NPs have also been detected in both cases. We assume that the partial agglomeration of NPs occurs during the evaporation of the solvent.

The elemental composition of the chitosan/Ag NPs foils was determined using EDX. The foil cast from the blank chitosan solution without AgNO_3_ was considered as a control. The results of EDX are presented in Table 1.

All the samples contain carbon, oxygen, and nitrogen, typical elements for the chitosan chemical structure. Both samples with Ag NPs demonstrate a clear signal of Ag corresponding to 0.7 at.% in the case of the liquid cathode and 1.2 at.% in the case of the liquid anode. The difference in atomic concentrations of Ag in composite foils confirms the earlier UV–VIS findings of the more efficient production of Ag NPs in the system with the liquid anode.

**Figure 6:**
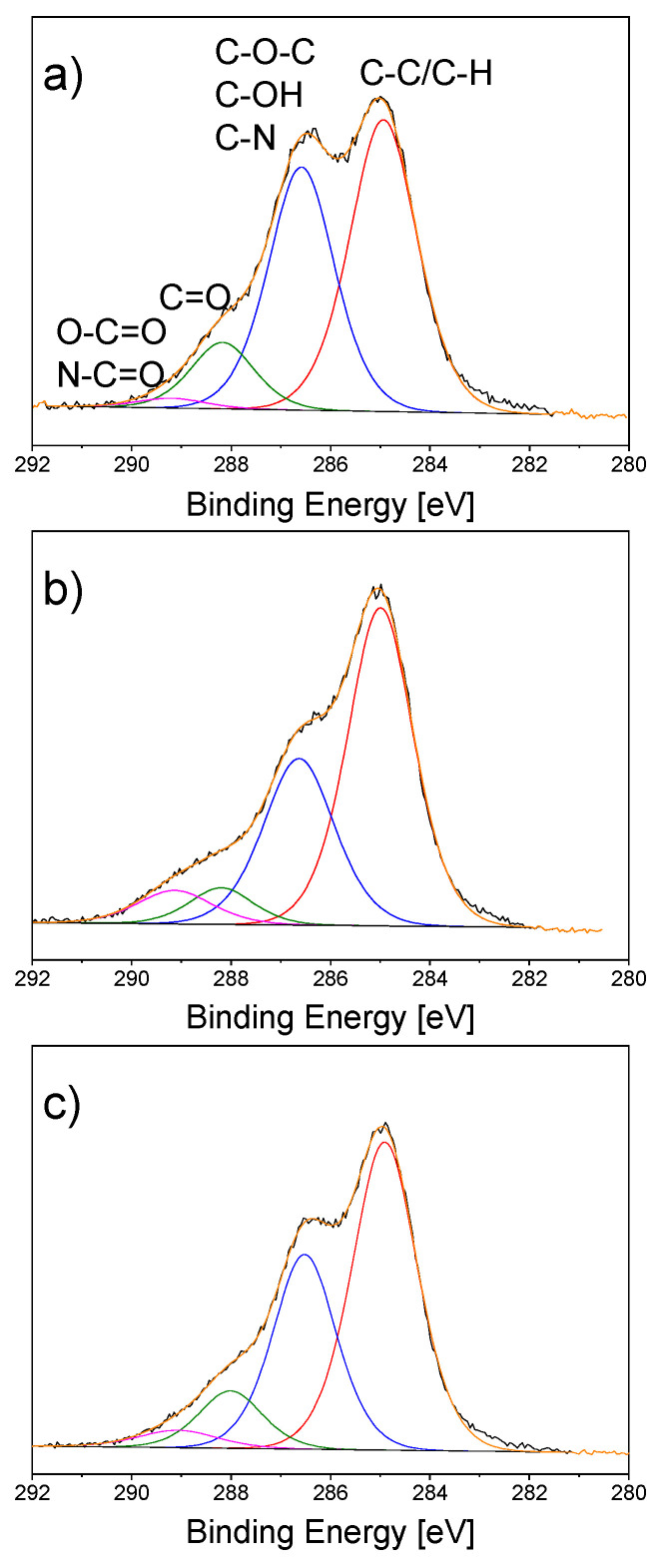
XPS C1s high-resolution spectra of (**a**) blank chitosan and chitosan/Ag NP composites prepared using a plasma-solution system with (**b**) the liquid anode, and (**c**) the liquid cathode. Treatment time, 30 min.

XPS was applied for the detailed analysis of chemical transformations that occurred with chitosan under the plasma action. Figure 6a shows the C1s high-resolution spectrum of blank chitosan foil.

The spectrum is rich in hydroxyl, ether, and primary amine groups, all these species contributing to an intensive signal at 286.5 eV. Smaller amounts of carbonyl (288.0 eV) and carboxyl (289.0 eV) groups are also present. Plasma-solution treatment of chitosan with both polarities of the liquid electrode (Figure 6b,c) results in a decrease of the peak at 286.5 eV that occurs due to the chitosan degradation confirming the partial cleavage of glucosidic chains. Moreover, the intensification of carbonyl and carboxyl group signals has been detected. This provides evidence of the glucoside bond destruction and side oxidation reactions.

**Figure 7:**
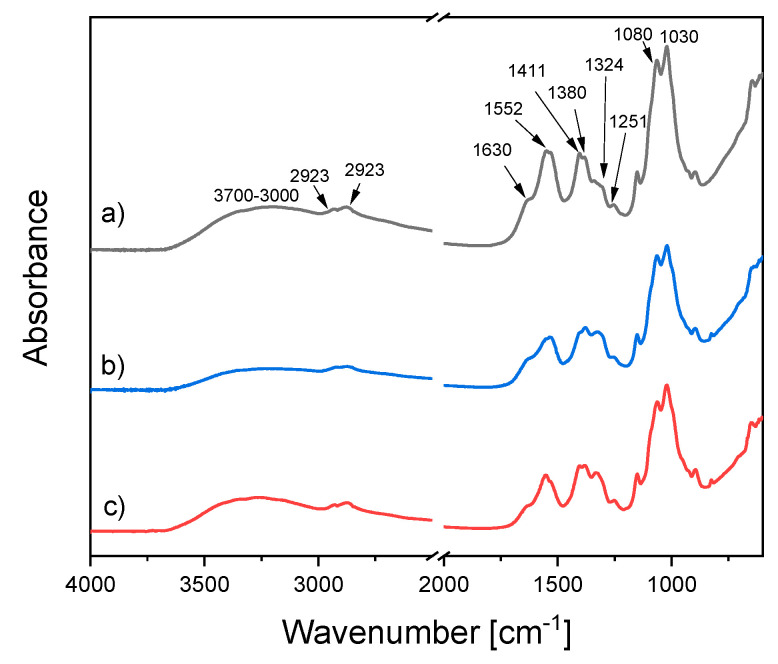
FTIR-ATR spectra of (**a**) blank chitosan and chitosan/Ag NP composites prepared using a plasma-solution system with (**b**) the liquid anode and (**c**) the liquid cathode. Treatment time, 30 min.

The possible chemical interaction of chitosan with Ag NPs can be characterized by FTIR spectroscopy. FTIR spectra of chitosan and chitosan/Ag NP composites prepared with both polarities of the liquid electrode are presented in Figure 7.

Here, a broad band in the region of 3700–3000 cm^−1^ is assigned to the stretching vibrations of hydroxyls and amines. Peaks at 2923 cm^−1^, 2877 cm^−1^, 1411 cm^−1^, 1324 cm^−1^, and 1251 cm^−1^ are associated with various vibrations of the CH_2_ groups. The peak at 1380 cm^−1^ corresponds to the CH_2_ wagging. The characteristic peaks at 1080 and 1030 cm^−1^ and of the subpeaks at 1160 and 900 cm^−1^ are related to the pyranose rings with the glycosidic linkages. It is worth noting that the intensity of bands at 1324 cm^−1^ and 1380 cm^−1^ grew after the plasma processing, which can be due to the chitosan degradation processes.

FTIR measurements are able to detect the possible interaction of chitosan with Ag in composites. According to the authors of [26], a difference in the relative intensity of amide bands can be observed due to the incorporation of Ag NPs into chitosan. The chemical interaction of Ag with chitosan manifests through the N–H bonds reducing their vibration intensity [26], the effect associated with an increase in the molecular weight of the Ag⋅⋅⋅N–H complex. Therefore, special attention should be paid to the peaks at 1552 cm^−1^ and 1630 cm^−1^ corresponding to free amine groups and acetylated amines, respectively. The integrated intensity ratio I_1552_/I_1630_ decreases if compared to that of blank chitosan so that I_1552_/I_1630_ is 1.6 and 1.9 for the liquid cathode and anode, respectively, which is lower than 2.3 corresponding to the blank chitosan. This result confirms the conclusion that Ag interacts with chitosan.

**Figure 8:**
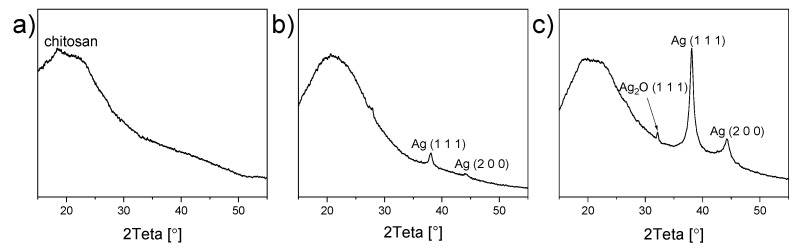
The XRD diffractograms of (**a**) blank chitosan and chitosan/Ag NP composites prepared using the discharge with (**b**) liquid cathode and (**c**) liquid anode. Treatment time, 30 min.

The XRD measurements were employed, and Figure 8 shows the diffractograms acquired for blank chitosan and chitosan/Ag NP composites.

The spectrum corresponding to blank chitosan shows a broad peak with a maximum of 2θ = 20°, related to the amorphous chitosan [20] (Figure 8a). This signal is also present in chitosan/Ag NP composites (Figure 8b,c). Two extra peaks appear at 38° and 44° in these diffractograms, and they can be attributed to the Ag crystalline phase in the orientation of (1 1 1) and (2 0 0). In agreement with the earlier data, the diffraction intensity is higher for the anode than the cathode, reflecting the higher concentration of Ag NPs in the former case. Surprisingly, a weak signal has also been detected at 32°, which does not belong to any metallic silver diffraction. This peak is seen on the diffractogram for the liquid anode and is most probably concealed by background for the liquid cathode. The diffraction can be assigned to the crystalline silver oxide Ag_2_O in the orientation of (1 1 1) [41].

### 3.3. Antibacterial Activity of Chitosan/Ag NP Composites

**Figure 9:**
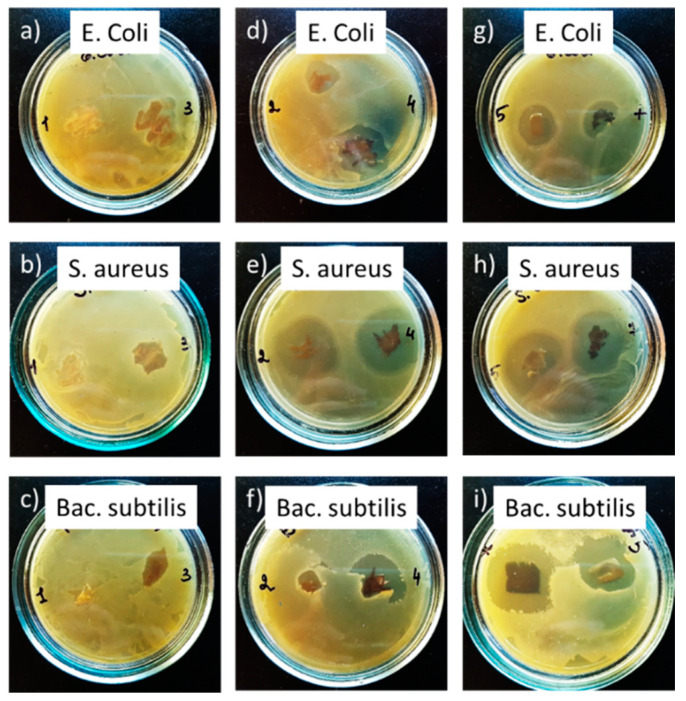
The antibacterial performance of (**a**–**c**) blank chitosan and chitosan/Ag NP composites prepared using the system with (**d**–**f**) liquid cathode and (**g**–**i**) liquid anode against *Escherichia coli*, *Staphylococcus aureus*, and *Bacillus subtilis*. Treatment time, 30 min. Incubation time, 24 h.

Possible biomedical applications of the prepared chitosan/Ag NP composites as elements of bandages or wound dressing materials require their strong antibacterial efficiency. Herein, the bactericidal effect of the foils was studied against the following three types of bacteria: Gram-negative *Escherichia coli*, Gram-positive *Staphylococcus aureus*, and Gram-positive *Bacillus subtilis*. The classic diffusion agar test was utilized, and the diameter of the zone of inhibition of bacteria growth was determined. The photos of agar plates with samples after 24 h of incubation are shown in Figure 9.

It can be seen that blank chitosan samples did not exhibit any bactericidal action. However, the clear zones of inhibition were formed around all the samples loaded with Ag NPs. Table 2 summarizes the results in terms of a normalized parameter of the antibacterial efficiency, which was calculated as the ratio of the area of inhibition zone to the sample area.

The most efficient antibacterial effect of composites was demonstrated against Gram-positive *Staphylococcus aureus*, whereas the worse performance was detected against Gram-negative *Escherichia coli* and Gram-positive *Bacillus subtilis*. It is known that Gram-positive bacteria are more susceptible to antibiotics than Gram-negative bacteria and easier to undergo destruction through the interaction with Ag^+^ ions [56]. In our case, we did not observe any correlation between the bactericidal action and the bacterial Gram response. Nevertheless, the bactericidal action difference was found for the liquid cathode against the liquid anode, with the latter demonstrating the superior bactericidal effect due to the higher content of Ag NPs (Table 1). Markedly, the most receptive *Staphylococcus aureus* does not show such a dependence, which is evidence that, in this case, the fast kinetics of the bactericidal action is not limited by the concentration of the bactericidal agent.

## 4. Discussion

Our group previously reported the degradation of chitosan under the action of plasma in the system with the liquid cathode [45,46]. The novelty of the present work is in the application of both possible polarities combined with the in situ formation of Ag NPs. The chitosan degradation rate constants for the discharge with the liquid cathode were found to exceed those for the opposite polarity. We attribute this effect to the different concentrations of the OH radicals that act as the primary reagents for the destruction of the chitosan chains. The formation of the OH radicals is a well-established fact for plasmas in or in contact with liquids. For the discharge with the liquid cathode, the OH radicals are formed more efficiently due to the positive ion bombardment of the solution surface. This statement is further supported by the measurements of the concentration of H_2_O_2_ in the solution, given that the reaction OH + OH → H_2_O_2_ is considered as the main channel for the recombination of the hydroxyl radicals in similar discharges. Indeed, the concentration of H_2_O_2_ in the system with the liquid cathode (4.90 × 10^-3^ mol/L) exceeds the concentration with the opposite polarity (1.15 × 10^-3^ mol/L).

The formation of oxidation agents during plasma treatment plays a key role in chitosan degradation. This effect can explain, for example, the appearance of the signal from the carbonyl groups in the UV–VIS spectra (see Figure 2a,b). Moreover, this signal grows more intensively for the liquid cathode due to a more efficient accumulation of the oxidation agents, such as H_2_O_2_ [57,58].

The formation of NPs in plasma-solution systems is mostly associated with the reduction of Ag^+^ ions by the solvated electrons according to the reaction Ag^+^ + e_aq_ → Ag^°^ [59]. The resulting atoms participate in the nucleation and growth under the van der Waals force action leading to the NP formation. Based on the UV–VIS measurements, it can be stated that Ag NPs are formed more effectively in the system with the liquid anode (Figure 2c,d). Here, the solution surface is bombarded by the electrons; therefore, the concentration of solvated electrons is higher, unlike the discharge with the liquid cathode.

The size of Ag NPs established from the TEM images (12 ± 5 nm in the system with the liquid cathode and 16 ± 6 nm in the system with the liquid anode) was found to be close to the sizes reported for other plasma-solution systems [49,50]. Nevertheless, in our case, we did not observe the agglomeration of NPs in solution detected by others [50]. The agglomerates of NPs appear in the composites’ structure, as seen in the SEM images. As mentioned above, the agglomerates are possibly formed during the evaporation of the solvent. It can be concluded that the surface of the composites is homogeneous and free from pores or cavities, at least at the scale of the SEM measurements.

It was intriguing to detect the signal from Ag_2_O in the XRD diffractogram because, typically, only metal Ag is reported both for conventional chemical synthesis [21,25,27,29,31,33] and non-equilibrium plasma-based approaches [49,50]. We assume the formation of Ag_2_O as a result of the accumulation of reactive oxygen species, such as atomic oxygen, hydroxyl radicals, or H_2_O_2_ in the solution under the plasma action. These species can interact with metallic Ag, partially oxidizing it with the formation of Ag_2_O. Such oxidized NPs can be perspective, for example, for the catalytic applications.

An essential aspect in the development of new composites is cost efficiency, especially in relation to the existing techniques. Our plasma-solution approach requires only three components, namely chitosan, acetic acid, and AgNO_3_, without the involvement of either additional catalysts or stabilizers. According to the author of [60], chitosan’s annual production is equal to 5 × 10^5^ kg with a price of 11.5 $/kg, which makes the consumables’ costs low. Considering the electric power of tens of watts and the preparation time of tens of minutes, we conclude that the plasma-solution method can be competitive for the fabrication of novel chitosan/Ag NP composites.

## 5. Conclusions

The plasma-solution approach with a liquid electrode (cathode or anode) is a facile and green technique for producing chitosan/Ag NP composite materials. The action of atmospheric pressure DC discharge initiates the simultaneous reduction of Ag^+^ toward the Ag NPs and the destruction of chitosan through the cleavage of glycoside bonds and side oxidation reactions. The degradation rate of chitosan in the presence of Ag^+^ ions is higher than the rate in their absence for both modes of the plasma-solution system. The principle difference between the two modes is related to the efficiency of Ag NP formation. The discharge with the liquid anode is more suitable for the preparation of chitosan/Ag NP composites due to faster NP growth. Nevertheless, Ag NPs of spherical shape and mean diameter of less than 20 nm were obtained for both types of electrode polarity. Composite chitosan/Ag NP foils are smooth and homogeneous, with random Ag NP distribution. The XRD analysis demonstrated the presence of Ag_2_O. The composites prepared with both polarities of the liquid electrode demonstrate a strong antibacterial effect against *Escherichia coli*, *Staphylococcus aureus*, and *Bacillus subtilis*.

## Figures and Tables

**Table 1:** Elemental composition of chitosan and chitosan/Ag NP foils determined by EDX.

Sample	C, at.%	O, at.%	N, at.%	Ag, at.%
chitosan	51.6	41.4	7.1	-
chitosan/Ag NPs, liquid cathode	48.3	42.6	8.5	0.7
chitosan/Ag NPs, liquid anode	48.4	42.2	8.1	1.2

**Table 2:** The bactericidal efficiency of chitosan and chitosan/Ag NP foils.

Sample	Area of the Inhibition Zone/Area of the Sample		
	*E. coli*	*Staphylococcus aureus*	*Bacillus subtilis*
chitosan	0	0	0
chitosan/Ag NPs, liquid cathode	4.1 ± 0.1	12.8 ± 0.2	4.3 ± 0.2
chitosan/Ag NPs, liquid anode	7.3 ± 1.2	11.8 ± 0.8	5.9 ± 0.1
